# A scoping review of risk factors for urinary tract infections following renal transplantation

**DOI:** 10.1186/s12894-026-02124-2

**Published:** 2026-03-26

**Authors:** Mingxi Kuang, Jiayuan Chen, Zhen Li

**Affiliations:** 1 School of Nursing, Kunming Medical University, Yunnan 650500 Kunming, China; 2 Department of Organ Transplantation, The First Affiliated Hospital of Kunming Medical University, Wuhua District, Kunming, Yunnan 650032 China

**Keywords:** Renal transplantation, Urinary tract infections, Risk factors, Scoping review, Evidence-based nursing

## Abstract

**Objective:**

This review aims to identify the risk factors associated with urinary tract infections(UTIs) after kidney transplantation, providing a reference for the early recognition and prevention of these infections in clinical practice.

**Methods:**

The Joanna Briggs Institute methodology framework for scoping reviews was adopted as the methodological guidance, and literature searches were performed in PubMed, Web of Science, the Cochrane Library, Embase, and CINAHL, with the search period spanning from the inception of each database to October 15, 2025. Data extraction and summary analysis were carried out on the included literature.

**Results:**

A total of 29 studies were included, and 28 risk factors for UTIs after kidney transplantation were identified, which were categorized into four groups: patient factors, disease-related factors, treatment-related factors, and other factors.

**Conclusions:**

Existing research indicates that factors influencing UTIs after kidney transplantation are multifaceted (including patient factors, disease-related factors, treatment-related factors, and other factors) and exhibit regional differences. It is necessary to control these influencing factors according to specific circumstances and to develop appropriate nursing measures.

## Introduction

Chronic kidney disease (CKD) refers to structural changes in the kidneys accompanied by a decline in renal function. Over time, some patients will progress to end-stage renal disease (ESRD), and kidney transplantation is widely recognized as the most effective treatment for this condition [1]. A study showed that the absolute number of kidney transplants performed in China reached 12,039 cases in 2021, representing a 9.1% increase compared with 2020 [2]. With the continuous advancement of surgical techniques and immunosuppressive regimens, the outcomes of kidney transplant recipients have been significantly improved. Nonetheless, UTI is the most common infectious complication after kidney transplantation, accounting for approximately 50% of all such complications [3]. UTI is defined as the pathological invasion of the urinary tract by infectious agents, which subsequently triggers an inflammatory response and the onset of corresponding clinical symptoms and signs [4]. According to the EAU guidelines on urological infections [5], UTIs are divided into two main categories: localized urinary tract infections (such as cystitis) and systemic urinary tract infections (such as pyelonephritis and prostatitis).The definition of localized urinary tract infections includes:1)Cystitis with typical signs/symptoms (e.g., frequency, urgency, suprapubic pain);2)No signs/symptoms of systemic infection;3)Applies to all sexes;4)Risk factors may be present and should be addressed.The definition of systemic urinary tract infections includes:1)UTI with signs/symptoms of systemic infection (e.g., fever, chills);2)May also include typical local symptoms (e.g., for pyelonephritis or prostatitis);3)Risk factors may be present and should be addressed. The incidence of UTIs following kidney transplantation ranges from 20% to 80% [6]. UTIs can increase the risk of further complications in transplant recipients, and are particularly associated with potential drug interactions, the development of antibiotic-resistant bacteria [7], impaired long-term graft survival, and even elevated mortality rates [8]. Numerous scholars [9–11] have focused on the research regarding risk factors for UTIs following kidney transplantation. However, these risk factors are complex and diverse, with variations across regions and individuals. Currently, there is a lack of literature summarizing the risk factors for UTIs after kidney transplantation. Although several meta-analyses have summarized UTIs after renal transplantation, there is a lack of a broader, JBI-style scoping review to depict the regional variability of risk factors and the knowledge map.A scoping review is a broader and more flexible knowledge synthesis of a specific research area, which can identify research achievements and gaps in the field and provide information support for further studies [12]. Although numerous original studies have been published since 2020, they have not been synthesized into a systematic scoping review, leaving a gap in our understanding of recent research hotspots and regional distribution.This study utilizes a scoping review methodology to clarify specific knowledge gaps in the field of risk factors for urinary tract infections after kidney transplantation. It connects these gaps with the advantages of the scoping review method and provides a concise research objective statement guided by the PCC framework, aiming to offer a reference for the early recognition and prevention of UTIs after clinical kidney transplantation.

## Methods

This study adopts the scoping review guidelines issued by the Joanna Briggs Institute (JBI) in Australia [13] as its methodological framework.The study was registered on the Open Science Framework (OSF) with the registration DOI: 10.17605/OSF.IO/3DWVF.

### Defining the research question

What are the risk factors for post-renal transplantation UTIs ?

### Inclusion and exclusion criteria for literature

According to the PCC principle [13]: participants (P), concept (C), and context (C) determine the inclusion criteria: [1] Participants: kidney transplant patients, aged ≥ 18 years; [2] Concept: the research topic focuses on risk factors for urinary tract infections after kidney transplantation (including localized urinary tract infections and systemic urinary tract infections); [3] Context: clinical environment following kidney transplantation; [4] Study types: randomized controlled trials, case-control studies, cohort studies, cross-sectional studies. Exclusion criteria: [1] Duplicated publications (only retaining the most comprehensive and up-to-date one); [2] Literature for which the full text is not accessible; [3] Non-English literature.

### Search strategy

A systematic search was conducted in PubMed, Web of Science, the Cochrane Library, Embase, and CINAHL by combining subject headings and free - text terms. Meanwhile, snowballing was performed on the references, and a search for gray literature was also carried out(including research registration platforms, institutional reports, and dissertation databases). The search period covered from the establishment of each database to October 15, 2025. The search strategy was as follows: (((((Kidney transplantation[MeSH Terms]) OR (Renal Transplantation[Title/Abstract])) OR (Kidney Grafting[Title/Abstract])) OR (solid organ transplant[Title/Abstract])) AND ((((Urinary tract infection[MeSH Terms]) OR (Infection, Urinary Tract[Title/Abstract])) OR (Tract Infection, Urinary[Title/Abstract])) OR (UTI[Title/Abstract]))) AND ((((Risk factors[MeSH Terms]) OR (Influence factors[Title/Abstract])) OR (predictive factors[Title/Abstract])) OR (factors[Title/Abstract])).We systematically searched major non-English databases relevant to the research topic, retrieving a total of 154 articles, with an omission rate of less than 10%.

### Literature screening and data extraction

The retrieved literatures were imported into EndNote 21 software and Covidence. After removing duplicates, two professionally trained researchers performed an initial screening by reading the titles and abstracts of the literatures in accordance with the inclusion and exclusion criteria, followed by a secondary screening through full-text reading of the eligible literatures. Two trained researchers independently extracted data using a self-designed Excel data extraction form developed after a literature review, including the study title, authors, publication year, country, sample size, incidence rate, and influencing factors. Literature screening and data extraction were conducted independently by two researchers (Mingxi Kuang and Jiayuan Chen, Master of Nursing). In case of discrepancies, a third researcher (Zhen Li, Master of Nursing, Deputy Chief Nurse, arbiter) was responsible for the final and independent review and judgment of the disputed literatures, and her decision was deemed final. Based on clinical practice and literature review, we classified the risk factors for urinary tract infections after kidney transplantation into four categories: patient factors, disease-related factors, treatment-related factors, and other factors.

### Evaluation of literature quality

Case-control and cohort studies were evaluated using the Newcastle-Ottawa Quality Assessment Scale (NOS) [14], which consists of three dimensions (eight items in total): selection of the study population, comparability between groups, and assessment of exposure or outcome, with a star scoring system (one point for each star). For cross-sectional studies, the Agency for Healthcare Research and Quality (AHRQ) scale [15] was used for quality assessment. The AHRQ scale contains 11 items in total, each scored as “yes” (1 point), “no” or “unclear” (0 points).For randomized controlled trials, the Cochrane Risk of Bias tool [16] was used for quality assessment. The assessment included the following seven items: random sequence generation, allocation concealment, blinding, completeness of outcome data, selective reporting of study results, and other sources of bias. Each item was judged by the researchers as “high risk of bias”, “low risk of bias”, or “unclear”.

## Results

### Results of literature screening

**Fig. 1:**
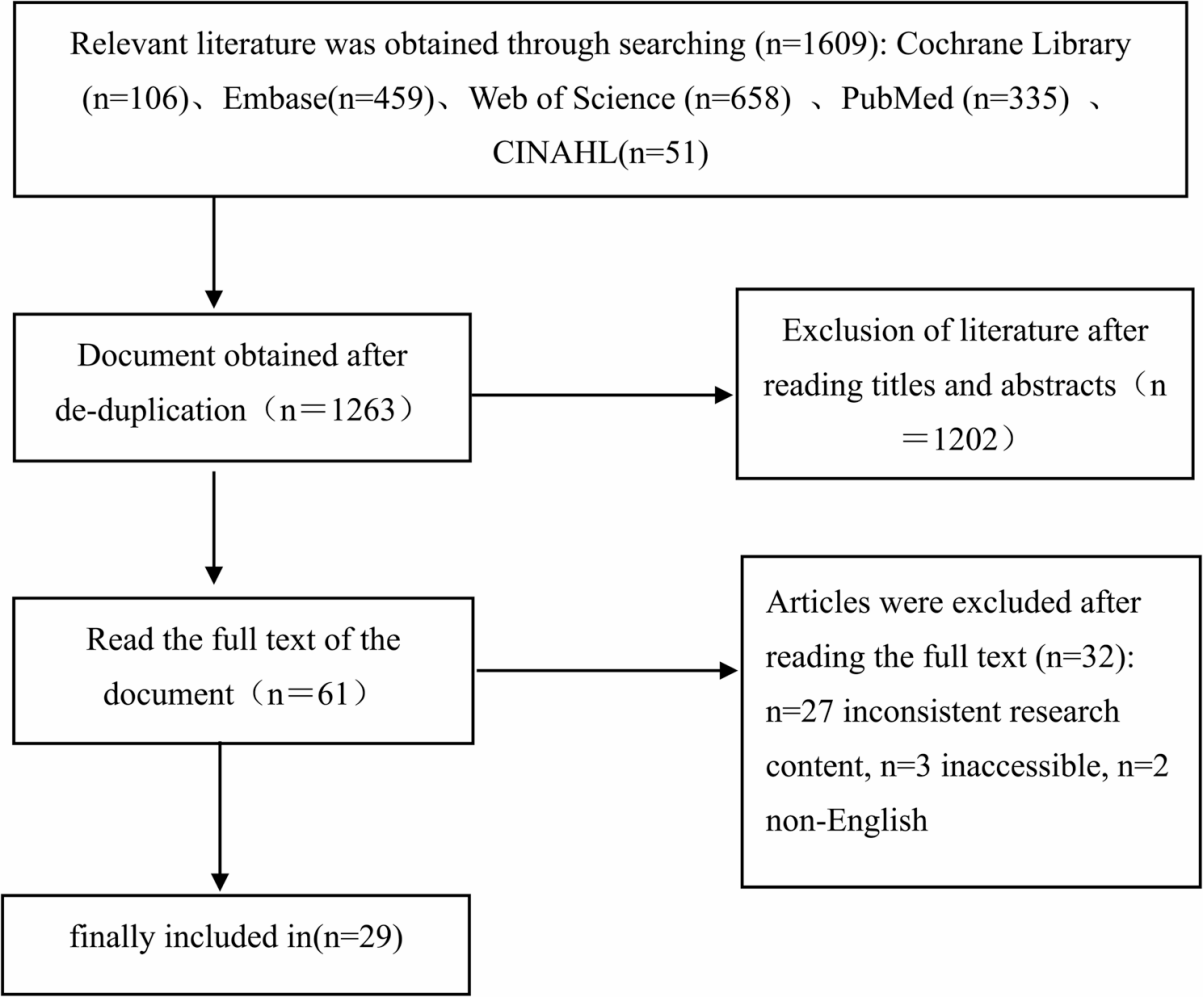
Literature screening flow chart

A preliminary search yielded 1,609 relevant studies, including 106 from the Cochrane Library, 459 from Embase, 658 from Web of Science, 335 from PubMed, and 51 from CINAHL. After removing duplicate records using EndNote 21 software and Covidence, 1,263 studies remained. A total of 1,202 studies that did not meet the inclusion criteria were excluded following a review of their titles and abstracts. Ultimately, 29 studies were included after full-text assessment [6, 9–11, 17–41]. The flow chart of literature screening is presented in (Fig. 1). 

### Basic characteristics of included literature

Among the 29 included studies, there is 1 randomized controlled trial [27], 2 cross-sectional studies [10, 22], 2 prospective cohort studies [28, 41], and 24 retrospective cohort studies [6, 9, 11, 17–21, 23–26, 29–40]. The total number of participants in the included studies is 7,797. The publication dates range from 2005 to 2024, with articles published after 2020 accounting for 51.72% (*n* = 15). The basic characteristics of the included studies are presented in Table 1.1.

**Table 1:** Literature characteristics (*n* = 29)

Year	Author	Title	Country	Sample	Incidence	Risk factors	Research type	Age and gender distribution	UTI Definition	UTI Assessment Time Period
2005	Chuang P [17]	Urinary tract infections after renal transplantation: a retrospective review at two US transplant centers	US	500	43%	Age, female, cadaveric donor	Retrospective cohort study	44 ± 12.6 331/169	③	42(6–78)months
2011	Papasotiriou M [18]	Predisposing factors to the development of urinary tract infections in renal transplant recipients and the impact on the long-term graft function	Greece	122	60.70%	Female, urinary system complications	Retrospective cohort study	44 ± 12 75/47	①	67.8 ± 30.5 months
2013	Lee JR [19]	Independent Risk Factors for Urinary Tract Infection and for Subsequent Bacteremia or Acute Cellular Rejection: A Single-Center Report of 1166 Kidney Allograft Recipients	US	1166	21%	Female, long-term use of Foley catheter, ureteral stent, age, and delayed graft function	Retrospective cohort study	N 714/452	①	3 months post-operation
2014	Camargo LF [20]	Urinary Tract Infection in Renal Transplant Recipients: Incidence, Risk Factors, and Impact on Graft Function	US	105	34.20%	Age, previous pregnancies, kidney sourced from expanded criteria donors (deceased donors), and acute rejection reaction	Retrospective cohort study	47.9 ± 11 67/38	N	One year post-operation
2014	Gołębiewska JE [21]	Urinary tract infections during the first year after renal transplantation: one center’s experience and a review of the literature	Poland	209	38%	Female, immune induction, history of recurrent UTI prior to RTx, acute reject reaction, CMV infection, urological complications	Retrospective cohort study	46.4 ± 14 124/85	①/②/⑧	1 months post-operation
2015	Gondos AS [22]	Urinary Tract Infection among Renal Transplant Recipients in Yemen	Yemen	150	33.30%	diabetes mellitus, urinary system complications	Cross-sectional study	N 93/57	N	N
2015	Kwon YE [23]	Vitamin D Deficiency Is an Independent Risk Factor for Urinary Tract Infections After Renal Transplants	South Korea	171	30.40%	vitamin D deficiency	Retrospective cohort study	40.8 ± 10.3 95/76	②	7.42 ± 2.20 Y
2015	Menegueti MG [24]	Study of the risk factors related to acquisition of urinary tract infections in patients submitted to renal transplant	Brazil	99	26.20%	Female	Retrospective cohort study	50(19–71) 57/42	④	One year post-operation
2016	Gozdowska J [25]	Urinary Tract Infections in Kidney Transplant Recipients Hospitalized at a Transplantation and Nephrology Ward: 1-Year Follow-up	Poland	107	23.50%	Female, impaired graft function, and a history of urinary tract intervention	Retrospective cohort study	52.51(21–71) 38/69	①	6 months post-operation
2017	Kotagiri P [26]	Urinary Tract Infections in the First Year PosteKidney Transplantation: Potential Benefits of Treating Asymptomatic Bacteriuria	Australia	276	57%	Female, double J stent	Retrospective cohort study	51 ± 13 185/91	②	One year post-operation
2017	Liu S [27]	Early Removal of Double-J Stents Decreases Urinary Tract Infections in Living Donor Renal Transplantation: A Prospective, Randomized Clinical Trial	China	103	29.40%	Double J stent indwelling time	Randomized controlled trial	N 79/24	①/⑦	3 months post-operation
2017	Mohan M [28]	Risk Factors for Urinary Tract Infections in Renal Allograft Recipients: Experience of a Tertiary Care Center in Hyderabad, South India	India	31	41.90%	Delayed graft function and prolonged hospital stay	Prospective cohort study	32.4 ± 10.2 24/7	⑤	Two year post-operation
2017	Ooms L [29]	Urinary Tract Infections After Kidney Transplantation: A Risk Factor Analysis of 417 Patients	Netherlands	417	28%	Female, recipient age > 60 years, surgical reintervention	Retrospective cohort study	55 ± 14 261/156	⑩	3 months post-operation
2019	Olenski S [30]	Urinary tract infections in renal transplant recipients at a quaternary care centre in Australia	Australia	72	27.80%	Female, age	Retrospective cohort study	N 38/34	①	Two year post-operation
2020	Hamid RB [31]	Multiple Drug Resistant Urinary Tract Infection in Kidney Transplant Recipients: A Retrospective Cohort Study	Pakistan	72	38.90%	Female, prolonged catheterization time, combined with diabetes mellitus, ATG induction therapy	Retrospective cohort study	48.4 ± 6.6 51/21	②	339 ± 125 d
2020	Ma ZZ [32]	Analysis of risk factors for early urinary tract infection after kidney transplantation	China	129	48.10%	Female and Delayed Graft Function	Retrospective cohort study	41.88 ± 10.75 85/44	⑥	1 months post-operation
2020	Tekkarışmaz N [33]	Risk Factors for Urinary Tract Infection After Kidney Transplant: A Retrospective Analysis	Turkey	145	37.90%	Female, glomerulonephritis as the primary renal disease, pre-transplant diabetes mellitus, and ureteral stent	Retrospective cohort study	35.2 ± 12.4 96/49	⑦	One year post-operation
2021	Arabi Z [34]	Urinary Tract Infections in the First 6 Months after Renal Transplantation	Saudi Arabia	279	35%	Age > 40 years, female, urinary system complications	Retrospective cohort study	43.4 ± 16 165/114	①/②/⑧/⑨	6 months post-operation
2021	Mosqueda AO [35]	Association Between the Placement of a Double-J Catheter and the Risk of Urinary Tract Infection in Renal Transplantation Recipients: A Retrospective Cohort Study of 1038 Patients	Mexico	966	19.60%	Double J stent	Retrospective cohort study	N 652/314	①	N
2021	Shimizu T [36]	Predictive factors and management of urinary tract infections after kidney transplantation: a retrospective cohort study	Japan	182	25.10%	Long-term postoperative malnutrition, voiding dysfunction and/or storage dysfunction after transplantation	Retrospective cohort study	N 117/65	②	1393(29-2534)day
2021	Velioglu A [6]	Incidence and risk factors for urinary tract infections in the first year after renal transplantation	Turkey	102	20.50%	Age, duration of indwelling urinary catheter, urological complications	Retrospective cohort study	37.6 ± 12.2 53/49	②	One year post-operation
2022	Koga S [11]	Influence of Graft Ureter Length, a Donor-Related Factor, on Urinary Tract Infections After Living-Donor Kidney Transplantation: A Single-Center Analysis of 211 Cases	Japan	211	13.70%	Length of transplanted ureter	Retrospective cohort study	N 143/68	①/②/⑧	40(11.5-445.5)d
2022	Nascimento EHG [37]	Effects of Bacterial Urinary Tract Infection on Clinical Outcome and Survival of Kidney Transplant Patients	Brazil	601	15.97%	Female, received DD kidney (deceased donor), rejection reaction episode and DGF	Retrospective cohort study	N 384/217	①/⑧	N
2022	Ozawa K [38]	Diabetes Mellitus as a Predictive Factor for Urinary Tract Infection for Patients Treated with Kidney Transplantation	Japan	236	14.0%	diabetes mellitus	Retrospective cohort study	47(38–59) 143/93	②	N
2023	Al Tamimi AR [39]	The Impact of Urinary Tract Infections in Kidney Transplant Recipients: A Six-Year Single-Center Experience	Saudi Arabia	553	41.59%	Female, history of bladder dysfunction	Retrospective cohort study	N 345/208	①/②/⑧/⑨	N
2023	Gala I [40]	A single-centre report of acute pyelonephritis in a patient after kidney transplantation – analyses of risk factors	Slovakia	153	30%	Female, diabetes mellitus, urinary system complications, acute rejection episode	Retrospective cohort study	49.04 ± 20.12 80/73	⑤	One year post-operation
2023	Shams SF [10]	Urinary Tract Infection in Renal Transplant Recipients: Incidence, Microbiological Profile and Predisposing Factors in India	India	432	31.25%	Female, urinary tract Parazacco spilurus subsp. spilurus often, prolonged hospitalization, hepatitis C virus infection	Cross-sectional study	37.2 ± 10 250/182	N	N
2024	Antonelli TS [41]	Body fat predicts urinary tract infection in kidney transplant recipients: a prospective cohort study	Brazil	67	23.90%	Waist circumference, visceral fat area, and high total fat mass	Prospective cohort study	43.6 ± 12.3 38/29	②	3 months post-operation
2024	Dziri S [9]	Prevalence and Predictive Factors of Urinary Tract Infection in Kidney Transplant Recipients: A 10-Year Study	Tunisia	141	51.10%	Female	Retrospective cohort study	32.54 ± 12.1 67/74	①	One year post-operation

N：Not mentioned, ①Asymptomatic bacteriuria: positive urine culture with >10⁵ CFU/mL bacteria, ②Uncomplicated UTI: positive urine culture with >10⁵ CFU/mL bacteria plus presence of symptoms (dysuria, urgency, frequency, suprapubic pain), ③Positive urine culture with >10⁵ CFU/mL bacteria plus ≥3 leukocytes per high-power field or >10⁵ leukocytes/mL in undiluted urine, ④Positive urine culture with >10⁵ CFU/mL bacteria plus presence of symptoms (dysuria, urgency, frequency, suprapubic pain) plus ≥3 leukocytes per high-power field or >10⁵ leukocytes/mL in undiluted urine, ⑤Presence of symptoms (dysuria, urgency, frequency, suprapubic pain), ⑥Presence of symptoms (dysuria, urgency, frequency, suprapubic pain) plus positive urine culture with >10⁵ CFU/mL bacteria or ≥3 leukocytes per high-power field or >10⁵ leukocytes/mL in undiluted urine (clinical diagnosis of UTI), ⑦≥3 leukocytes per high-power field or >10⁵ leukocytes/mL in undiluted urine, ⑧Complicated UTI: positive urine culture with >10⁵ CFU/mL bacteria plus presence of symptoms (fever, graft pain, chills, malaise), ⑨Recurrent UTI: ≥1 episode of UTI within the first 6 months, ⑩UTI was defined according to the following criteria: (1) urine culture with ≤2 microbial species (CDC), (2) at least 1 bacterial pathogen with >10⁵ CFU/mL (CDC), (3) treated with antibiotics (non-CDC), (4) occurred within 3 months after kidney transplantation (KT) (non-CDC)

### Evaluation results of literature quality

The quality evaluation results of cohort studies are presented in Table 2, those of cross-sectional studies are shown in Table 3, and those of randomized controlled trials are given in Table 4.

**Table 2:** Results of quality evaluation for cohort studies

Study	Selection of the study population	Inter-group comparability	Result measurement	Score
Chuang P	****	**	**	8
Papasotiriou M	****		***	7
Lee JR	****	*	**	7
Camargo LF	**	**	***	7
Gołębiewska JE	***	*	***	7
Kwon YE	***	**	***	8
Menegueti MG	****	*	***	8
Gozdowska J	****	*	***	8
Kotagiri P	****	*	***	8
Mohan M	****		***	7
Ooms L	****	*	***	8
Olenski S	****	*	**	7
Hamid RB	****		***	7
Ma ZZ	****	*	***	8
Tekkarışmaz N	****		***	7
Arabi Z	****	*	***	8
Mosqueda AO	****		**	6
Shimizu T	****		***	7
Velioglu A	***	**	***	8
Koga S	***	*	**	6
Nascimento EHG	****	*	**	7
Ozawa K	****	*	***	8
Al Tamimi AR	****		***	7
Gala I	***	**	***	8
Antonelli TS	***	*	***	7
Dziri S	****	*	***	8

Note: Each "*" counts one point

**Table 3:** Results of quality evaluation for cross-sectional studies

Study	Item											Score
	1	2	3	4	5	6	7	8	9	10	11	
Gondos AS	yes	No	yes	yes	yes	yes	No	No	No	yes	No	6
Shams SF	yes	No	yes	yes	unclear	yes	No	yes	No	No	No	5

**Table 4:** Quality evaluation results of randomized controlled trials

Study	Item						
	1	2	3	4	5	6	7
Liu S	low risk	low risk	low risk	unclear	low risk	low risk	low risk

### Risk factors for UTIs after renal transplantation

**Fig. 2:**
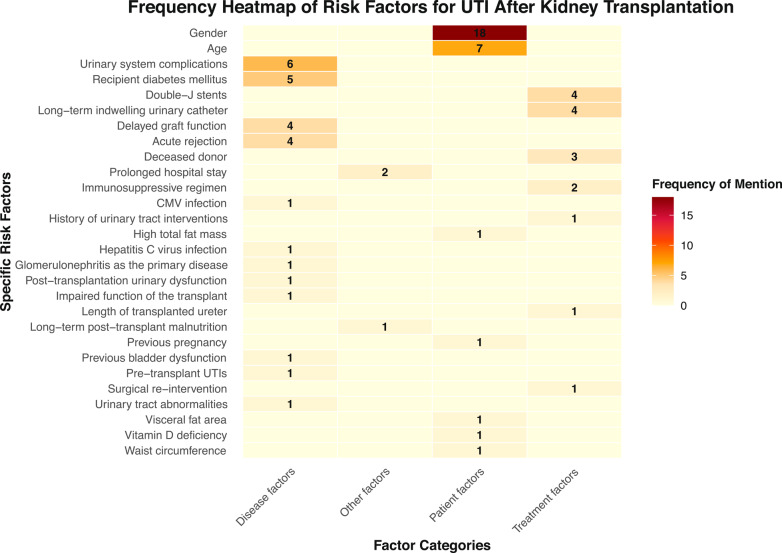
Frequency heat-map by factor category

A thematic analysis was conducted on the literature related to the influencing factors of UTIs after kidney transplantation. Based on clinical practice and literature review, we classified the risk factors for UTIs following kidney transplantation into four categories: patient factors, disease-related factors, treatment-related factors, and other factors, which included 7 patient factors, 12 disease-related factors, 7 treatment-related factors, and 2 other factors.We have drawn frequency heat maps based on four categories, as shown in (Fig. 2). Eleven factors have been reported with a frequency of ≥ 2 times: gender [9, 10, 17–19, 21, 24–26, 29–34, 37, 39, 40], duration of urinary catheterization [6, 19, 31], urological complications [6, 18, 21, 22, 34, 40], age [6, 17, 19, 20, 29, 30, 34], recipient diabetes [22, 31, 33, 38, 40], acute rejection [20, 21, 37, 40], duration of Double-J stent placement [19, 26, 27, 33, 35], delayed graft function [19, 28, 32, 37], deceased donor [17, 20, 37], prolonged hospital stay [10, 28], and immunosuppressive regimen [21, 31]. 

#### Patient factors

Patient factors mainly include gender [9, 10, 17–19, 21, 24–26, 29–34, 37, 39, 40], age [6, 17, 19, 20, 29, 30, 34], vitamin D deficiency [23], previous pregnancy [20], waist circumference and visceral fat area and high total fat mass [41]. Eighteen studies [9, 10, 17–19, 21, 24–26, 29–34, 37, 39, 40] showed that gender is a risk factor for UTIs after renal transplantation, with females being more susceptible. Age is positively correlated with the occurrence of UTIs; the older the age, the higher the incidence of UTIs [6, 17, 19, 20, 29, 30, 34].

#### Disease factors

The primary disease and post-transplant disease status are closely associated with the occurrence of UTIs after renal transplantation. Studies have shown that recipient diabetes mellitus [22, 31, 33, 38, 40], pre-transplant UTIs [21], glomerulonephritis as the primary renal disease [33], hepatitis C virus infection [10], and previous bladder dysfunction [39] are risk factors for UTIs following renal transplantation. Six studies [6, 18, 21, 22, 34, 40] indicated that post-transplant urinary system complications (such as kidney stones and hydronephrosis) can lead to the development of UTIs, while seven studies [19–21, 28, 32, 37, 40] showed that acute rejection and delayed graft function after renal transplantation are risk factors for UTIs.

#### Treatment factors

UTIs after renal transplantation are associated with the immunosuppressive regimen [21, 31] and deceased donor [17, 20, 37]. A Retrospective Cohort Study [31] showed that induction therapy with antithymocyte globulin (ATG) is highly correlated with the occurrence of UTIs. Seven studies [6, 19, 26, 27, 31, 33, 35] indicated that long-term indwelling urinary catheters and double-J stents are risk factors for UTIs. UTIs are closely associated with deceased donors [17, 20, 37].

#### Other factors

Both prolonged hospital stay [10, 28] and long-term post-transplant malnutrition [36] are high-risk factors for UTIs after renal transplantation.

## Discussion

### Differences in the incidence of UTIs

This article summarizes the 29 included studies, finding that the incidence of urinary tract infections after kidney transplantation ranges from 13.7% [11] to 60.7% [18]. The significant variations in the incidence of urinary tract infections after kidney transplantation reported in different studies may be attributed to differences in factors such as country, region, healthcare conditions, diagnostic criteria, and sample size.We performed a pooled analysis of the urinary tract infection incidence from 29 studies using a random-effects model in RevMan. The results showed that the pooled incidence of urinary tract infection after renal transplantation was 30% (95% CI: 26%–35%).

### Regional comparison

This study further compared the regional characteristics of studies on UTI after renal transplantation between Asia (9 studies: South Korea 1, India 2, Pakistan 1, China 2, Japan 3) and Western Europe (5 studies: Greece 1, Poland 2, Netherlands 1, Slovakia 1) listed in Table 1. Significant heterogeneity was identified between the two regions in terms of incidence, risk factors, and study design.Studies from Western Europe had a larger average sample size (234 cases vs. 174 cases in Asia) and a significantly higher average UTI incidence rate (37.7% vs. 30.3% in Asia). All 5 Western European studies used a retrospective cohort design with more consistent assessment criteria and time frames. In contrast, Asian studies exhibited greater diversity in study design, including randomized controlled trials and prospective cohorts, and showed wider intra-regional variation in incidence rates (13.7%–48.1%).Regarding risk factors, studies in Western Europe reached 100% consensus on female gender as a risk factor, and frequently focused on immune-and infection-related factors such as urinary system complications, acute rejection, and CMV infection. In Asia, only 33.3% of studies mentioned female gender; instead, they focused more on technical factors including duration of double‑J stent placement and length of the transplanted ureter. In addition, vitamin D deficiency and postoperative malnutrition were unique risk factors in this region. Furthermore, the timing and definition of UTI assessment were highly consistent in Western Europe, whereas standardized definitions were lacking in Asia. These differences may be related to regional diagnosis and treatment strategies, population characteristics, and study design. This suggests that in clinical prevention and control, Asia should strengthen screening for immune-related risk factors, while Western Europe can optimize technical details of transplantation procedures. Global UTI management should take into account both common risk factors and region-specific needs.

### Risk factors for UTIs after renal transplantation

#### Patient factors

Gender is the most frequently reported influencing factor in the included literature, with females being more susceptible than males. Firstly, anatomically, females have a shorter urethra that is closer to the anus, making them more vulnerable to *Escherichia coli* invasion, which can lead to ascending infections [42]. Secondly, compared with males, the urinary proteome of females is more prone to induce bacterial proliferation and inflammatory responses in urine [43]. A Proteomic Analysis [44] results show that the highly expressed urinary proteins in males were mainly those specific to the male urinary system, such as prostate-origin proteins, beta-microseminoprotein, prostate-specific antigen, and prostatic acid phosphatase. In contrast, the proteins that are highly expressed in the female urinary protein group are mainly related to fat and carbohydrate metabolism, including carbonic anhydrase 1, APOA4, APOC3, and lipocalin-1. These differences may reveal distinct mechanisms by which gender influences susceptibility to urinary tract infections. Furthermore, this study [44] indicated that the female urinary proteome exhibited greater inter-individual variability than that of males, which may be closely related to physiological processes such as cell movement, cell migration, and inflammatory responses. This suggests that these differences may exist prior to the occurrence of infection, thereby influencing an individual’s susceptibility to urinary tract infections. In future clinical practice, these proteins could be considered as preventive biomarkers to identify high-risk patients and implement early intervention measures.

Age is also one of the important influencing factors for urinary tract infections after kidney transplantation. As age increases, the immune system capacity of kidney transplant recipients gradually declines. Additionally, when comorbid conditions such as prostate enlargement and bladder atrophy are present, patients are more prone to urinary retention, thereby increasing the risk of UTIs [4]. Multiple studies have indicated that age > 40 years [34], > 60 years [29], and > 65 years [11, 35] are independent risk factors for UTIs. While there is still some controversy regarding this factor in current research, but the findings show a common trend that older patients are more susceptible to UTIs. Future large-sample, multi-center longitudinal studies could be conducted to further investigate this issue.

Vitamin D, as a potent immune modulator, can inhibit macrophages from releasing excessive inflammatory cytokines, promote macrophage maturation and lysosomal enzyme secretion, and induce the production of antimicrobial peptides (cathelicidin and β-defensin) [45]. An animal study has shown that when urothelial cells come into contact with bacteria, they rapidly produce and secrete cathelicidin. The epithelial-derived cathelicidin plays a significant protective role against urinary tract infections [46].

During pregnancy, the enlargement of the uterus compresses the pelvic floor muscles, which weakens their contraction strength and leads to a decline in the function of the urethral sphincter. As the bladder is compressed and pushed forward and upward, this elongates the urethra and significantly reduces bladder tone. After childbirth, the relaxation of the abdominal muscles in postpartum women can result in weakened or insufficient contraction of the bladder detrusor muscle and impaired micturition dynamics [47].

Obesity can lead to immune response dysregulation, manifested as impaired chemotaxis, altered macrophage differentiation, an imbalance in cytokine production, and imbalanced interactions between the immune system and adipocytes. Therefore, one major reason for urinary tract infections in obese individuals may be the dysregulation of the immune system and its unbalanced interactions with fat cells [48, 49]. Another reason is the reduced bactericidal activity of polymorphonuclear cells, which is associated with decreased activity of TLR4 receptors on their cell membranes [50]. Additionally, in male obese transplant recipients, another factor accounting for the higher incidence of urinary tract infections may be the increased prostate volume induced by obesity [51, 52].

#### Disease factors

After kidney transplantation, patients may experience a series of urinary tract-related complications, such as urinary fistula, vesicoureteral reflux, stricture, postoperative hydronephrosis, and lithiasis. Among these, urinary fistula is a common complication that mostly occurs in the lower segment of the ureter of the transplanted kidney. If the urinary fistula fails to be effectively managed, it may further induce UTIs [53].

Higher levels of glucose in urine provide an ideal environment for bacterial growth, promoting rapid bacterial proliferation [54]. Additionally, diabetic patients often have immune function deficiencies and may also experience neurogenic bladder and urinary retention due to neuropathy [55], which creates a favorable environment for bacterial growth in the bladder. Most studies [22, 31, 33, 38, 40] suggested that a history of diabetes increases susceptibility to UTIs in kidney transplant recipients. However, a retrospective review [17] indicated that diabetes may have no substantial effect on the occurrence of UTIs in patients after kidney transplantation.

A prospective cohort study has shown [27] that acute rejection can lead to fibrosis of the kidney graft, thereby increasing the risk of UTIs. Clinically, patients often require stronger immunosuppressive therapy to treat acute rejection, which further reduces their immunity and consequently increases the risk of UTI infection [56, 57].

Delayed graft function(DGF) is an independent risk factor for UTIs in kidney transplant recipients. The potential mechanisms may involve that DGF patients require a longer duration of polyclonal antibody immunotherapy and higher doses of antibodies. Polyclonal antibodies can induce apoptosis, complement-mediated cell death, and antibody-dependent cellular cytotoxicity. Excessive use of these antibodies can lead to an imbalance in immune mechanisms, thereby increasing the risk of infections [58]. A review has confirmed that increased use of polyclonal antibodies is closely associated with a higher risk of infection in post-transplant recipients [58]. However, a Retrospective Cohort Study has suggested that there is no significant correlation between DGF and the development of UTIs [35]. Further in-depth research is required to clarify its underlying mechanism in the future. When cytomegalovirus (CMV) infection occurs clinically, patients’ innate immunity is impaired, and CMV infection can weaken the host immune response, thereby predisposing individuals to infections [21].

The liver is a vital metabolic organ, and its dysfunction (e.g., liver cirrhosis) may impair renal function. Hepatitis C virus (HCV) constitutes one of the major causes of liver cirrhosis. Owing to the impaired immune function in cirrhotic patients, they are predisposed to bacterial infections, including UTIs [59, 60].

#### Treatment factors

The risk of UTIs in kidney transplant recipients is affected by the duration of urinary catheter indwelling. The longer the indwelling time, the more medical and nursing procedures are required. Patients may face an increased risk of UTIs due to factors such as healthcare providers failing to strictly adhere to aseptic techniques during operations, and untimely replacement of urinary catheters and urine collection bags. However, there is currently no consensus on the specific duration of urinary catheterization in relation to the incidence of UTIs. Study has indicated that a catheterization duration greater than 5 days is an independent risk factor for UTIs following kidney transplantation from donation after cardiac death (DCD) donors [61]. Future research needs to expand the study population and conduct multicenter, large-sample clinical studies.

The use of ureteral stents in kidney transplantation has been a topic of controversy. They can increase the safety of the ureterovesical anastomosis, prevent obstruction caused by edema or external compression of the ureter, and reduce the risk of ureteral torsion [35]. However, prolonged placement of double-J ureteral stents may lead to an increased incidence of postoperative UTIs. This is attributed to several factors, including interference with the ureter’s anti-reflux mechanism, promotion of calcium salt deposition in the renal pelvis, and the invasive nature of the stent removal process [62]. Currently, there is still controversy regarding the duration of Double-J stent placement. A study has indicated that leaving a Double-J stent in place for > 4 weeks increases the risk of UTIs [63] (OR, 2.79; 95% CI, 1.4–5.55). Furthermore, placing a Double-J stent for < 2 weeks does not effectively reduce the incidence of UTIs [64]. Even reports have indicated that UTIs can be resolved within one week postoperatively [27, 65], with the incidence of UTIs decreasing from 24.6% to 7.6% [66].

The immunosuppressant regimen is also one of the risk factors for UTIs after kidney transplantation. Controversies exist regarding the impact of specific immunosuppressant types on UTIs. A studies has suggested that recipients taking tacrolimus are more susceptible to UTIs. The specific mechanism remains unclear, but it may be associated with the stronger immunosuppressive effect of tacrolimus [62]. Other review has suggested that the use of antithymocyte globulin (ATG) is associated with a higher incidence of UTIs after kidney transplantation, while calcineurin inhibitors (e.g., tacrolimus) do not appear to affect the risk of UTIs [4]. ATG can suppress the T-cell immune function of the human body. Therefore, when used in the short term, it can alleviate ischemia-reperfusion injury of the transplanted kidney and reduce the incidence of DGF. However, due to its strong immunosuppressive properties, recipients are more susceptible to UTIs [67].

A Prospective study conducted by Rivera-Sanchez R et al. reported that the prevalence of UTIs in patients receiving kidneys from DCD donors was 70%, compared to 28% in those receiving kidneys from living donors [68]. A meta-analysis [69] suggested that kidneys from DCD donors are an independent risk factor for the occurrence of UTIs. This may be due to the prolonged ICU stay of DCD donors before death, which increases the likelihood of infections. in addition, the donor kidneys have a prolonged warm ischemia time and relatively poor quality. A retrospective study has indicated that due to pre-existing infections in DCD (donation after circulatory death) donors, the clinical practice may escalate the level of antibiotic prophylaxis post-surgery. As a result, the use of kidneys from DCD donors does not significantly affect the incidence of UTIs in recipients [70]. Notably, these studies do not mention the causes of death for DCD donors, leaving unclear whether the donors experienced accidental deaths while being otherwise healthy. Therefore, future research could focus on the impact of different causes of death in DCD donors and types of donation (DCD and DBD) on post-transplant infections. Most patients with end-stage renal disease receive dialysis replacement therapy before transplantation, and the long-term accumulation of metabolites can lead to immune dysfunction. Thus, pre-transplant dialysis is also an important risk factor for UTIs.

#### Other factors

The kidney is one of the primary organs responsible for regulating the acid-base balance of the human body. When the body’s acid-base balance regulatory mechanism is disrupted, resulting in alkaline urine, it can facilitate bacterial proliferation and elevate the risk of UTI development [71]. In addition, prolonged malnutrition, characterized by deficiencies in nutrients such as proteins, can impair the body’s immune system and reduce its resistance to infections [36]. After kidney transplantation, recipients need to take immunosuppressants for life to reduce the risk of rejection and protect graft function, which results in decreased immune capacity. A cohort study [28] has shown that a hospital stay longer than 10 days significantly increases the incidence of UTIs in kidney transplant recipients; the longer the hospital stay, the higher the risk of developing UTIs.

In conjunction with the key recommendations for the prevention and control of UTI risk factors in kidney transplant recipients outlined in the 2019 American Society of Transplantation (AST) Infectious Diseases Community Practice Guidelines on urinary tract infections, the clinical implications of this study are further supported by evidence.The guidelines clearly state that the prevention and control of risk factors for UTI after renal transplantation should consider both general and transplant-specific factors to form a stratified prevention and control system: Among general factors, female sex, advanced age, diabetes mellitus, and indwelling catheter/ureteral stenting are strongly associated risks that require routine clinical screening.Transplant-specific factors include acute rejection, deceased donor source, and induction therapy (antithymocyte globulin). These factors are directly related to post-transplant immune status and surgical procedures and are the key targets for prevention and control.The guidelines specifically emphasize the standardized management of transplant-related procedures: ureteral stent placement increases the risk of UTI by 49%, and early removal (within 2 weeks) can significantly reduce the risk of infection. This suggests that clinical practice should optimize the duration of stent indwelling and avoid unnecessary prolongation.

### Limitation

The types of studies included in this research are limited, with most being retrospective cohort studies. Additionally, only English-published articles were incorporated, which may have an impact on the generalizability of the results.

## Conclusion

This systematic review included 29 studies, covering 7,797 patients, and systematically identified 28 factors related to the outcome of urinary tract infections after kidney transplantation: patient factors, disease factors, treatment factors, and other factors. Clinical healthcare workers can refer to the findings of this study to achieve early identification and prevention of UTIs following kidney transplantation. Based on the above analysis of risk factors, the following suggestions are proposed(such as Table 1.1): [1] Precise patient intervention: For high-risk groups such as the elderly, females, and those with underlying urinary system diseases or diabetes, complete preoperative evaluations including urine routine and urine culture; strengthen health education and guide patients to drink 1500-2000 ml of water daily, urinate frequently, and keep the perineum clean [2]. Optimization of diagnosis and treatment procedures: Strictly control the indications for invasive operations; rationally use immunosuppressants and antibacterial drugs to avoid the abuse of immunosuppressive agents and broad-spectrum antibacterial drugs; promptly intervene in postoperative complications such as calculi and obstruction [3]. Improvement of medical management: Establish a risk scoring system to realize preoperative stratification and postoperative precise monitoring; construct a multidisciplinary collaboration model involving the Department of Organ Transplantation and the Department of Infectious Diseases to improve the quality of whole-course management.

However, there is still some controversy regarding the risk factors for UTIs after kidney transplantation. Future studies should involve large sample sizes and longitudinal or interventional designs to further verify the causal relationships of these risk factors. At the same time, it is important to explore targeted intervention measures and evaluate the effectiveness of different preventive strategies through randomized controlled trials, providing higher levels of evidence for clinical practice.

## Data Availability

The data in this study is not sensitive in nature and is accessible in the public domain. The data is therefore available and not of a confidential nature. The data that support the findings of this study are available from the corresponding author upon reasonable request.
